# Association of Maternal Prepregnancy Body Mass Index With Fetal Growth and Neonatal Thalamic Brain Connectivity Among Adolescent and Young Women

**DOI:** 10.1001/jamanetworkopen.2020.24661

**Published:** 2020-11-03

**Authors:** Marisa N. Spann, Dustin Scheinost, Tianshu Feng, Kristiana Barbato, Seonjoo Lee, Catherine Monk, Bradley S. Peterson

**Affiliations:** 1 Columbia University Irving Medical Center, New York, New York; 2 Yale University School of Medicine, New Haven, Connecticut; 3 New York State Psychiatric Institute, New York; 4 Children’s Hospital Los Angeles, Los Angeles, California; 5 Department of Psychiatry, Keck School of Medicine, University of Southern California, Los Angeles

## Abstract

**Importance:**

Higher maternal prepregnancy body mass index (BMI; calculated as weight in kilograms divided by height in meters squared) is associated with adverse long-term outcomes for offspring, including obesity, poorer cognitive and social abilities, and increased risk of psychiatric disorders. Less clear is whether higher maternal BMI disrupts fetal growth and brain development.

**Objective:**

To investigate the association of maternal prepregnancy BMI with fetal growth and neonatal functional connectivity.

**Design, Setting, and Participants:**

This prospective longitudinal cohort study was conducted from 2012 to 2017. Participants included nulliparous pregnant adolescent and young adult women, aged 14 to 19 years who were recruited in the second trimester through Columbia University Irving Medical Center and Weill Cornell Medical College. Women received routine prenatal care and had no major health problems at the time of recruitment. Data were analyzed from January 2018 to March 2020.

**Exposures:**

Maternal prepregnancy BMI.

**Main Outcomes and Measures:**

The main outcomes were fetal growth, measured as estimated fetal weight, and neonatal functional connectivity, measured using magnetic resonance imaging. Prepregnancy BMI and fetal ultrasonographic measurements were obtained from electronic health record review. Resting-state brain imaging data were acquired in infants within the first month of postnatal life. Functional connectivity was measured using intrinsic functional distribution and seed-based methods.

**Results:**

Among 129 women recruited, 105 had ultrasonographic data from at least 2 points and were included in analyses. The mean (SD) age at delivery was 17.82 (1.31) years. Maternal prepregnancy BMI was positively associated with the slope of estimated fetal weight (β = 0.668; 95% CI, 0.163 to 1.175; *P* = .01) but not with fetal head circumference (β = −0.004; 95% CI, −0.024 to 0.016; *P* = .70). In a subsample of 45 infants with magnetic resonance imaging data, maternal prepregnancy BMI was positively correlated with global connectivity in the left thalamus. Using this thalamic region as a seed, higher maternal BMI was associated with greater local thalamic (both hemispheres) and lower frontothalamic connectivity.

**Conclusions and Relevance:**

These results suggest that maternal prepregnancy BMI was associated with the development of regulation of body weight and thalamic functional brain connectivity in offspring even during fetal development.

## Key Points

**Question:**

Is maternal prepregnancy body mass index among adolescent and young women associated with fetal growth and neonatal functional connectivity?

**Findings:**

In this cohort study that included 129 pregnant adolescent and young adult women, maternal prepregnancy body mass index had a significant positive association with the slope of estimated fetal weight and with greater local thalamic and lower frontothalamic connectivity.

**Meaning:**

These findings suggest that maternal prepregnancy body mass index was associated with the regulation of body weight and functional connectivity of the thalamus in newborn infants.

## Teaser

This cohort study examines the association of prepregnancy BMI with fetal growth development and functional connectivity outcomes among adolescent and young women.

## Introduction

In the United States, 37% of women of childbearing age have overweight or obesity.^1^ Higher prepregnancy body mass index (BMI; calculated as weight in kilograms divided by height in meters squared), occurring mainly in the context of prepregnancy or gestational diabetes, has been associated with worse cognitive development, socialization, and verbal communication in offspring from preschool to school age.^2,3,4,5^ The association of maternal prepregnancy obesity with increased risk in offspring of psychiatric disorders, including attention-deficit/hyperactivity disorder and autism spectrum disorder, has been inconsistent.^6,7,8^ Nevertheless, findings from nonhuman primate and rodent studies of fetuses exposed to maternal high-fat diets and thereby to increased maternal weight include reduced attention and increased appetite, anxiety, hyperactivity, and aggression in offspring.^9,10^ The findings suggest that maternal BMI before or during pregnancy has significant long-term effects on developing offspring. However, very few studies consider the early antecedents of poor future outcomes,^11^ at a time when prevention is more feasible, despite widespread recognition that prenatal or fetal programming and fetal adaptations to the environment, play an integral role in future disease risk. Our study seeks to determine whether prepregnancy BMI is associated with fetal growth and brain development as an essential step to fill this gap in knowledge.

Exposure to higher maternal BMI has been associated across species with increased fetal weight.^12,13^ The few studies in humans that considered higher maternal BMIs and fetal growth parameters, including weight, head circumference, fat adiposity, and abdominal circumference, report mixed findings.^14,15^ However, these studies relied on comparing mothers with overweight or obesity with mothers with weight within reference range. Collapsing data at the extremes of a weight distribution largely ignores nuances in the associations of individual variability with maternal BMI values on characteristics of the developing fetus. A complete distribution of maternal BMI is needed to fully characterize these possible fetal outcomes. Many prior studies have not been able to disentangle the influence of maternal BMI from cooccurring health-related (eg, diabetes, hypertension) factors,^16^ which is important to help inform preventive efforts.

Studies are beginning to use advanced imaging methods, such as ultrasonography and magnetic resonance imaging (MRI), to associate measurements of maternal BMI with the developing brain. For example, maternal prepregnancy and pregnancy BMI are associated with greater fetal head circumference, as an estimate of total brain volume,^17^ across pregnancy in women with obesity, compared with women with weight within reference range.^18,19^ Additionally, neonates of mothers with overweight or obesity compared with those of mothers with weight in reference range, have been reported to have altered (decreased or increased) MRI-based functional connectivity measures across multiple brain regions.^20,21^ While these findings can be largely attributed to prenatal rather than postnatal exposures, further prospective longitudinal studies of the developing human brain are needed from fetal through early postnatal development.

To address these limitations of previous studies, we acquired fetal ultrasonographic data retrospectively and functional MRI data prospectively in neonates (ie, from birth to age 6 weeks) born to a large sample of pregnant adolescent and young adult women who had a full range of prepregnancy BMIs. The goal of our study was to understand the association of maternal prepregnancy BMI with early functional brain development and how fetal growth measures may be associated with mediating the association of prepregnancy BMI measures with infant brain outcomes. We hypothesized that prepregnancy BMI would be positively associated with the slope of fetal weight and head circumference measures. We also hypothesized that higher maternal prepregnancy BMI would correlate bidirectionally with measures of neonatal functional connectivity in brain regions supporting attention and regulatory control (ie, dorsal medial prefrontal cortex, cingulate, hypothalamus, or thalamus), consistent with prior reports.^20,21^

## Methods

This cohort study was approved by the institutional review board of the New York State Psychiatric Institute. All participants provided written informed consent in person. This study is reported following the Strengthening the Reporting of Observational Studies in Epidemiology (STROBE) reporting guideline.

### Participants

Nulliparous pregnant adolescent and young adult women aged 14 to 19 years were recruited in their second trimester through the Departments of Obstetrics and Gynecology at Columbia University Irving Medical Center and Weill Cornell Medical College and via flyers posted in the vicinity as part of a longitudinal study examining adolescent pregnancy behaviors and infant outcomes. Participants were excluded if they acknowledged use of tobacco or medications that could affect cardiovascular functioning (eg, α blockers, β blockers), corticosteroids, or chronic-use asthma medications or lacked fluency in English.

### Procedures

#### Prepregnancy BMI and Pregnancy Weight Gain

Prepregnancy BMI was calculated based on height and weight information provided by the pregnant participants. Maternal self-report of prepregnancy weight has a high level of accuracy for purposes of calculating BMI, and the minimal underestimations that occur do not heavily impact BMI categorizations.^22,23^ We considered 4 categories of maternal BMI, according to the international classification: underweight was defined as BMI less than 18.5; reference range, BMI 18.5 to less than 25; overweight, BMI 25 to less than 30; and obesity, BMI 30 or greater.^24^ To ensure that our findings regarding prepregnancy BMI did not derive primarily from other maternal factors that have been associated with fetal growth, we controlled for maternal weight gain and metabolic or health complications during pregnancy.^25,26^ Pregnancy weight gain, an index of overall health during pregnancy,^27^ was also obtained by weighing pregnant women in their second and third trimester study visits.

#### Electronic Health Record Review

Prenatal electronic health records (EHRs) were reviewed to determine maternal health conditions, including prepregnancy or gestational diabetes and hypertension, as well as other pregnancy complications, such as infection and fetal distress. These data were dichotomized depending on whether the mother did or did not have the condition. Gestational age at birth, birth weight, Apgar score, and delivery data were also determined from the EHR. Gestational age at birth was determined from the EHRs for dates of ultrasonographic examinations and last reported menstrual cycle.

Fetal morphometric measures were obtained from ultrasonographic records. They included head circumference, femur length, biparietal diameter, and abdominal circumference. Head circumference was measured along the outer margin of the calvaria at the level of the biparietal diameter. Femur length was measured between the proximal and distal metaphysis. Biparietal diameter was measured from the outer margin of the proximal skull table to the inner margin of the distal skull table. Abdominal circumference was measured along the outer boundaries of the abdomen at the level of the portoumbilical vein complex and thoracic vertebrae T10 to T11. We chose fetal weight and head circumference as the primary fetal outcomes for the study because fetal weight provides an index of nutrient intake and is calculated using multiple growth parameters and because head circumference provides an estimate of brain growth and is strongly correlated with intelligence at school age.^17^ Estimated fetal weight (EFW) was determined by combining the biparietal diameter, femur length, and abdominal circumference data in the Hadlock formula 30: *log*(*EFW*) = 1.3596 − 0.00386 × *AC* × *FL* + 0.0064 × *HC* + 0.00061 × *BPD* × *AC* + 0.0424 × *AC* + 0.174 × *FL*, in which *AC* indicates abdominal circumference; *FL*, femur length; *HC*, head circumference; and *BPD*, biparietal diameter.^28^ For growth modeling of EFW and head circumference, only participants with 2 or more ultrasonograph records were included in analyses.

### Imaging Procedures

#### Infant Scanning

Infants were scanned within the first weeks of postmenstrual life (ie, the time elapsed between the first day of the pregnant women’s last menstrual period and the time of the MRI scan of their infant). Infants were fed, swaddled, and acclimated to the scanning environment and scanner noise by listening to a tape recording of the scanner sounds played before each pulse sequence. They were given time to fall asleep, without the use of sedatives, while lying on the scanner bed before the start of each sequence. Foam and wax ear plugs, along with ear shields (Natus Medical), were applied to dampen scanner noise. MRI-compatible electrocardiographic leads were placed on the infant’s chest, and a pulse oximetry sensor was placed on the infant’s toe. Heart rate and oxygen saturation were continually monitored during the scan (In Vivo Research). Images were obtained using a 3 Tesla Signa MRI scanner (General Electric) and an 8-channel head coil. Near the middle of the study’s data collection, the MRI scanner was upgraded. Details on pulse sequences can be found in the eAppendix in the Supplement.

#### Common Space Registration

First, anatomical images were skull stripped using FSL software (FMRIB Analysis Group). All further analyses were performed using BioImage Suite^29^ unless otherwise specified. Anatomical images were linearly aligned to a single infant anatomical scan from an independent study^30^ using a 12-parameter affine registration that maximized the normalized mutual information between images. Next, anatomical images were nonlinearly registered to an evolving group mean template in an iterative fashion using a previously validated algorithm.^31^ This algorithm iterates between estimating a local transformation to align individual brains to a group mean template and creating a new group mean template based on the previous transformations. The local transformation was modeled using a free-form deformation parameterized by cubic B-splines. This transformation deforms an object by manipulating an underlying mesh of control points. The deformation for voxels between control points was interpolated using B-splines to form a continuous deformation field. Positions of control points were optimized using a conjugate gradient descent to maximize the normalized mutual information between the template and individual brains. After each iteration, the quality of the local transformation was improved by increasing the number of control points and decreasing the spacing between control points to capture a more precise alignment. A total of 5 iterations were performed with decreasing control point spacings of 15 mm, 10 mm, 5 mm, 2.5 mm, and then 1.25 mm. To help prevent local minima during optimization, a multiresolution approach was used with 3 resolution levels at each iteration. Finally, functional images were rigidly aligned to the corresponding anatomical images. All transformation pairs were calculated independently and combined into a single transformation, warping the single participant results into common space. This single transformation allows the individual participant images to be transformed to the common space with only 1 transformation, thereby reducing interpolation error.

#### Connectivity Processing

Motion correction was performed using SPM software version 8 (Wellcome Center for Human Neuroimaging). Images were warped into 3 mm^3^ common space using the nonlinear transformation and cubic interpolation. Next, images were iteratively smoothed until the smoothness of any image had a full-width half maximum of approximately 8 mm using the 3dBlurToFWHM package of AFNI software (National Institutes of Health). This iterative smoothing reduces motion-related confounds.^32^ Several covariates of no interest were regressed from the functional MRI time series, including linear and quadratic drifts, mean cerebrospinal fluid signal, mean white matter signal, and mean gray matter signal. For additional control of possible motion-related confounds, a 24-parameter motion model (including 6 rigid-body motion parameters, 6 temporal derivatives, and these terms squared) was regressed from the data. The functional data were temporally smoothed with a Gaussian filter (approximate cutoff frequency, 0.12 Hz). Only voxels in the gray matter were used in further calculations.

#### Intrinsic Functional Connectivity

After preprocessing, functional connectivity of each voxel, as measured by intrinsic connectivity distribution, was calculated for each infant as described previously.^33^ Intrinsic connectivity distribution involves correlating the time series for any voxel with the time series for every other voxel in the brain or brain hemisphere and then calculating a summary statistic based on the network theory measure degree. Intrinsic connectivity distribution avoids the need for choosing an arbitrary connectivity threshold by modeling the entire distribution of correlation thresholds using a Weibull distribution: βα(*r*α)β − 1exp(−(*r*α)β), in which *r* is a correlation between 2 time series, *α* is the variance parameter, and *β* is the shape parameter. This parameterization is akin to modeling the change in network theory metric degree with a stretched exponential as the threshold used to calculate degree is increased: exp(−τβa), in which *τ* is the correlation threshold, and *α* is the variance parameter, and *β* is the shape parameter. Specifically, the time series for any gray matter voxel was correlated with every other voxel in the gray matter. A histogram of these correlations was constructed to estimate the distribution of connections to the current voxel. This distribution was converted to a survival function, and the survival function was fitted with a stretched exponential with unknown variance. As variance controls the spread of the distribution of connections, a larger variance indicates a larger number of high correlation connections. Finally, this process was repeated for all voxels in gray matter, resulting in a whole-brain parametric image summarizing the degree of connectedness of each tissue element.

#### Seed Connectivity

Post hoc seed connectivity analysis was performed to explore the nodes identified by intrinsic connectivity distribution analysis to determine the specific connections that were most responsible for changes in connectivity. Seeds were defined on the reference brain and transformed back (via the inverse of the transforms) into individual infant space. The time course of the reference region in a given infant was then computed as the mean time course across all voxels in the reference region. This time course was correlated with the time course for every other voxel in gray matter to create a map of *r* values, reflecting seed-to–whole brain connectivity. These *r* values were transformed to *z* values using Fisher transform, yielding 1 map for each seed and representing the strength of correlation with the seed for each infant.

### Statistical Analysis

To assess the associations of the variable of interest with potential confounding variables, demographic and behavioral data were analyzed using standard χ^2^ test statistics or Fisher exact test for categorical data. Continuous data were analyzed using *t* tests or Mann-Whitney *U* tests when a normal distribution could not be assumed to compare groups. Associations of fetal growth with prepregnancy BMI were derived using the slope of EFW and head circumference values across 2 or more ultrasonographic time points. The slope was then associated with prepregnancy BMI in a linear regression model. All analyses were performed using SPSS statistical software version 25 (IBM) or SAS statistical software version 9.3 (SAS Institute). *P* values were 2-sided, and *P* < .05 was considered statistically significant.

Imaging data were analyzed using intrinsic connectivity distribution, voxel-wise linear models controlling for sex, postmenstrual age, and scanner upgrade, with all 3 covariates included in a single model. Significance was assessed at *P* < .05, with all maps corrected for multiple statistical comparisons across gray matter using cluster-level correction estimated via the 3dClustSim package version 16.3.05 in AFNI with 10 000 iterations, an initial cluster forming threshold of *P* = .001, the gray matter mask applied in preprocessing, and a mixed-model spatial autocorrelation function. Parameters for the spatial autocorrelation function were estimated from the residuals of the voxel-wise linear models using 3dFWHMx.

We performed path analysis to explore the association of prepregnancy BMI with fetal growth indices and neonatal brain connectivity. For this analysis, prepregnancy BMI was the independent variable, fetal growth was the variable potentially associated with mediation, and neonatal brain connectivity was the dependent variable. An indirect mediation association was tested using bootstrapping (5000 iteration), which does not assume a normal distribution of mediation associations. Mediation analyses were performed using the PROCESS macro for SPSS.^34^

## Results

### Demographic Characteristics

Of the 129 participants recruited, 110 (85.3%) had ultrasonographic data, and 105 (81.4%) had these data for at least 2 points. Among offspring of recruited mothers, 72 newborns underwent MRI scanning, and 45 newborns (62.5%) had usable functional and anatomical MRI data. The mean (SD) age of the pregnant women was 17.82 (1.31) years, and a large proportion (94 women [89.5%]) were Hispanic individuals (Table). Most infants were delivered vaginally (82 deliveries [82.8%]), all were appropriate size for gestational age (mean [SD] birth weight, 3191.18 [485.95] g; mean [SD] gestational age at birth, 39.4 [1.3] weeks), and 56 infants (53.3%) were boys. Very few delivery complications were noted (18 deliveries with complications [18.2%]). Postnatally, the infants were scanned at a mean (SD) postmenstrual age of 42.5 (1.7) weeks. Demographic characteristics for the total sample with ultrasonographic data did not differ significantly from the subsample with MRI data. The mean (SD) prepregnancy BMI was 25.8 (7.2), with a wide distribution of BMIs, ranging from 14.42 to 52.52. Only 1 mother had a diagnosis of diabetes, and her BMI was within the reference weight range. There was no significant association between prepregnancy BMI and pregnancy weight gain.

**Table.** Maternal and Neonatal Demographic Characteristics

Characteristic	No. (%)	
	Sample with ultrasound data (n = 105)	Subsample with MRI data (n = 45)
**Maternal**		
Age at delivery, mean (SD), y	17.82 (1.31)	17.71 (1.38)
Prepregnancy BMI, mean (SD)	25.78 (7.15)	25.11 (6.52)
Education, grade		
8th	1 (1.0)	0
9th	10 (10.0)	5 (11.1)
10th	10 (10.0)	7 (15.6)
11th	30 (30.6)	9 (20.0)
≥12th	47 (48.0)	21 (46.7)
Race/ethnicity		
Not Hispanic/Latino	11 (10.5)	6 (13.3)
Hispanic/Latino	94 (89.5)	39 (86.7)
Type of delivery		
Vaginal	82 (82.8)	31 (68.9)
Cesarean	17 (17.2)	9 (20.0)
Pregnancy complications^a^		
None	81 (81.8)	32 (71.1)
Any	18 (18.2)	7 (15.5)
**Neonatal**		
Gestational age at birth, mean (SD), wk	39.39 (1.31)	39.25 (1.37)
Birth weight, mean (SD), g	3191.18 (485.95)	3155.40 (438.30)
Birth head circumference, mean (SD), cm	33.89 (1.28)	33.96 (1.45)
Apgar, mean (SD)		
1 min	8.33 (1.39)	8.63 (0.81)
5 min	8.78 (0.90)	8.95 (0.22)
Postmenstrual age at scan, mean (SD), y	NA	42.46 (1.71)
Gender		
Male	56 (53.3)	31 (68.8)
Female	49 (46.7)	14 (31.1)

Abbreviations: BMI, body mass index (calculated as weight in kilograms divided by height in meters squared); NA, not applicable.

^a^ Includes chorioamnionitis, group B streptococcus, and acute nephritic syndrome.

### Main Analyses

**Figure 1.**
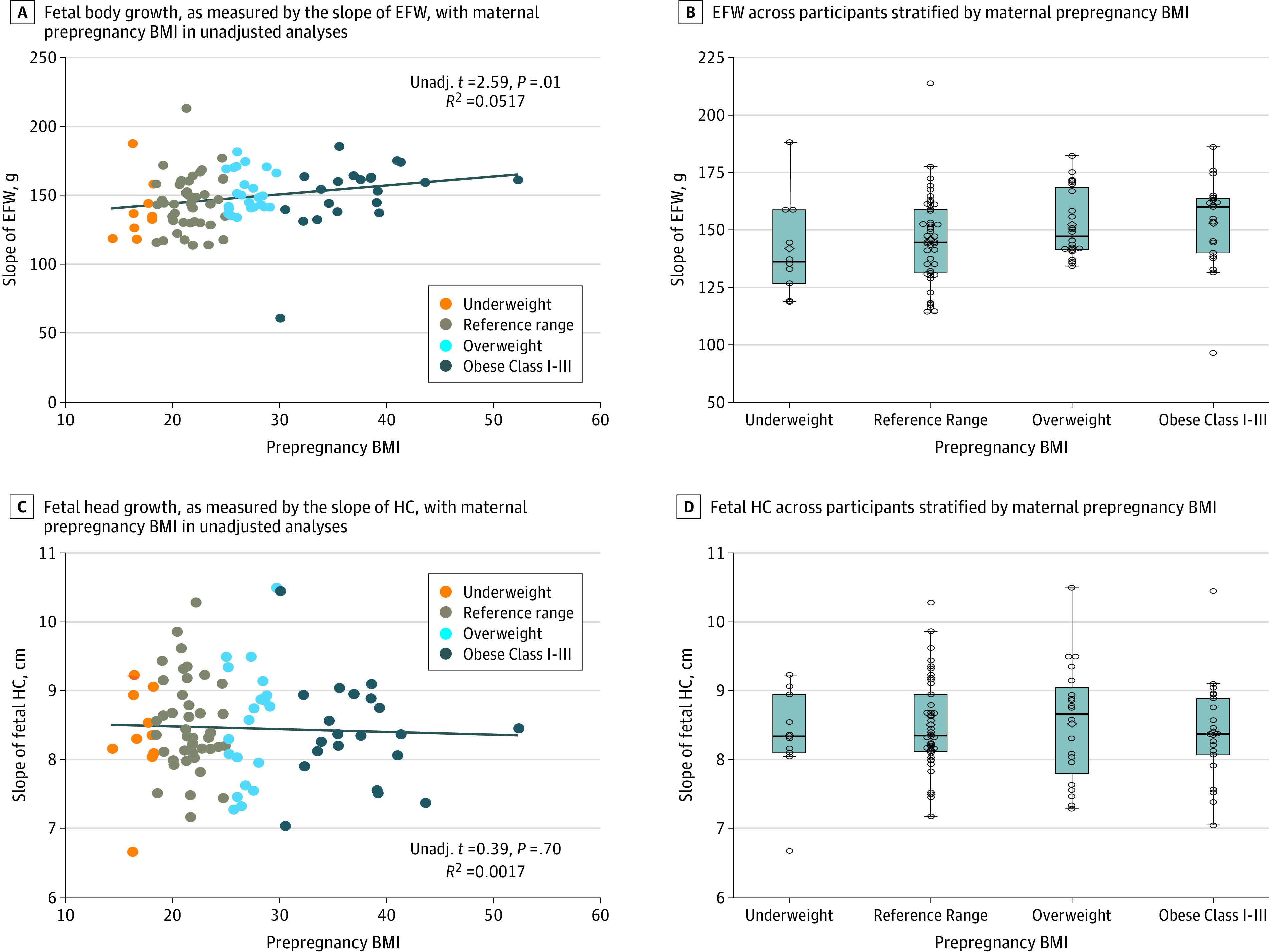
Associations of Prepregnancy Body Mass Index (BMI) With Growth Curve of Estimated Fetal Weight (EFW) and Fetal Head Circumference (HC) A and C, Dots indicate individual data points; line, trend. Unadj. indicates unadjusted. B and D, Lines indicate median; boxes, interquartile range; whiskers, the minimum and maximum excluding any outliers; dots, potential outliers.

Higher maternal prepregnancy BMI was significantly associated with a greater rate of change (ie, accelerated) fetal growth, as measured by EFW (β = 0.668; 95% CI, 0.163-1.175; *P* = .01) (eTable in the Supplement). Figure 1A displays a scatterplot showing the distribution of the EFW and prepregnancy BMI across the 4 BMI classes. The data are well-distributed across the BMI classes and demonstrate a linear association, suggesting that acceleration of fetal weight gain increases proportionally with maternal prepregnancy BMI. Models adjusting for pregnancy weight and complications as well as maternal demographic variables remained significant (eTable in the Supplement).

On consideration of sex differences in the association of prepregnancy BMI and EFW, we found that for boys, higher maternal prepregnancy BMI was significantly associated with greater EFW (*r* = 0.321; 95% CI, 0.063 to 0.537; *P* = .02). There was no association for girls (*r* = 0.165; 95% CI, −0.126 to 0.422; *P* = .27) or for the interaction of BMI with sex (*z* = 0.84; 95% CI, −0.88 to 1.21; *P* = .40).

Higher maternal prepregnancy BMI was not significantly associated with greater fetal head circumference in unadjusted or adjusted models (eTable in the Supplement) or when stratified by sex (boys: *r* = −0.150; 95% CI, −0.397 to 0.117; *P* = .30; girls: *r* = 0.081; 95% CI, −0.205 to 0.353; *P* = .60). Figure 1B displays a scatterplot showing the distribution of fetal head circumference and BMI across the 4 BMI categories.

**Figure 2.**
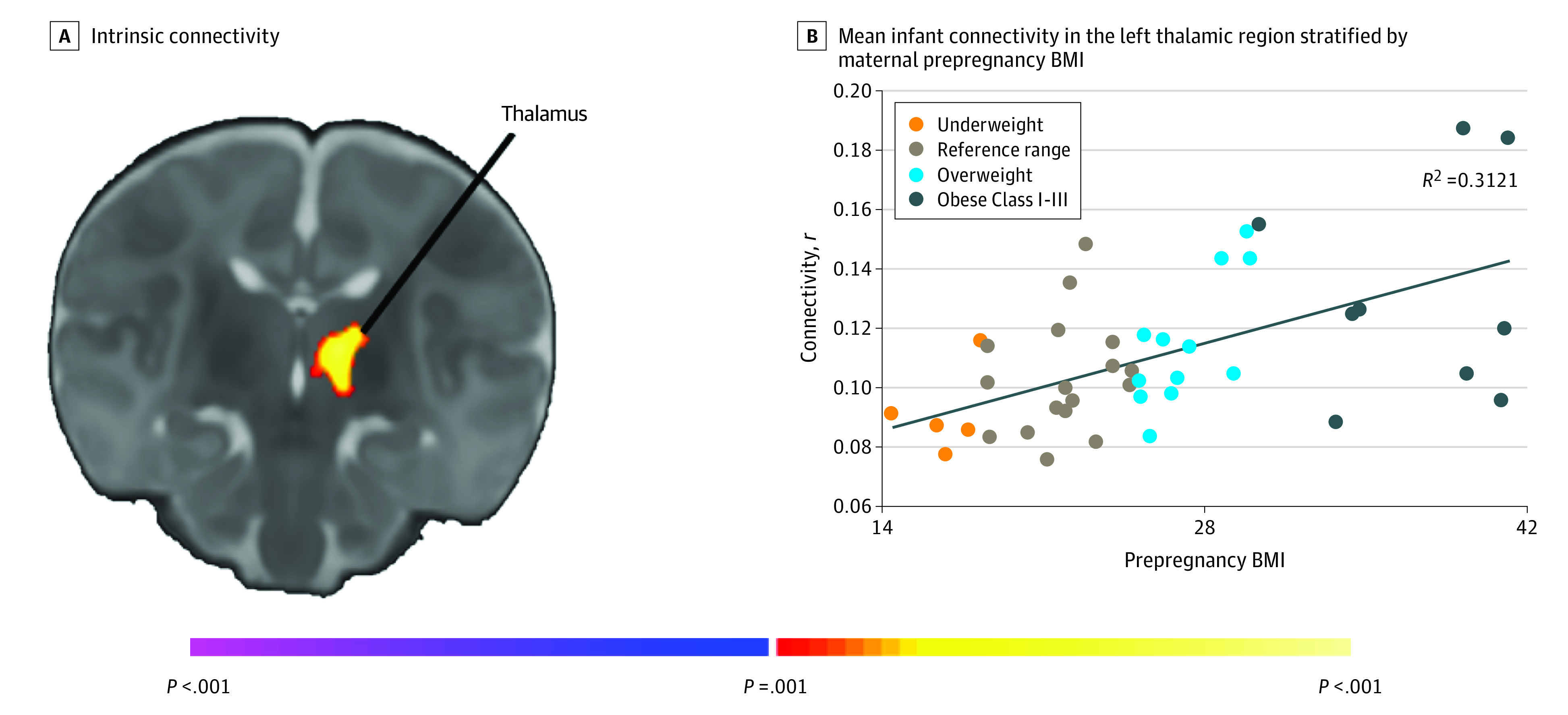
Associations of Prepregnancy Body Mass Index (BMI) and Whole Brain Connectivity Higher maternal prepregnancy BMI using the intrinsic connectivity distribution was associated with stronger connectivity between the left thalamus and the whole brain. The scatterplot visualizes the distribution of the observed data points for mean infant connectivity in the left thalamic region plotted against prepregnancy BMI.

**Figure 3.**
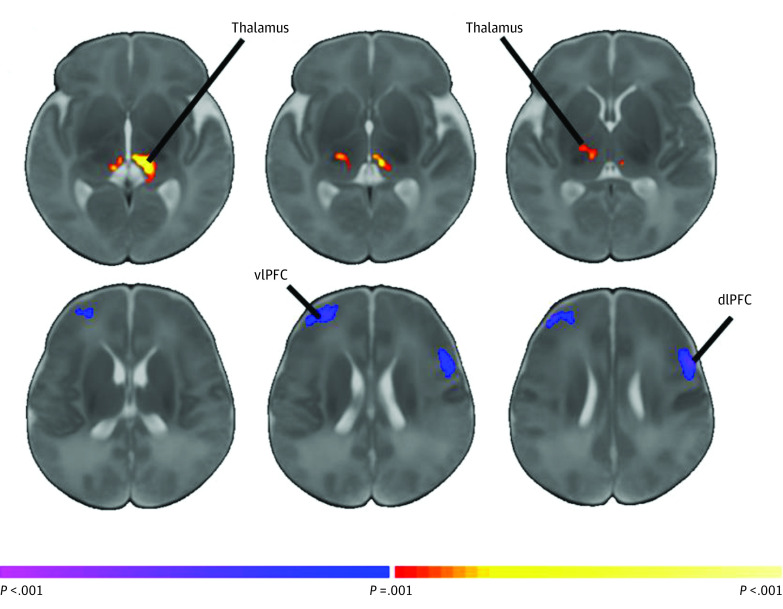
Thalamic Seed-Based Connectivity The left thalamic seed demonstrates greater local connectivity with the bilateral thalamus. The thalamic seed also demonstrated reduced connectivity with the right ventrolateral prefrontal cortex (vlPFC) and left dorsolateral prefrontal cortex (dlPFC) regions.

A significant positive correlation of prepregnancy BMI with global connectivity in the left hemithalamus was observed, such that higher BMI was associated with greater connectivity of the left thalamus to the whole brain (Figure 2). On further analysis using the left thalamic region as a seed, we found that higher maternal BMI was associated with increased local thalamic connectivity, and with decreased right ventrolateral frontothalamic and left dorsolateral frontothalamic connectivity (Figure 3). These models controlled for child sex, postmenstrual age at scan, and scanner.

### Post Hoc Analyses

**Figure 4.**
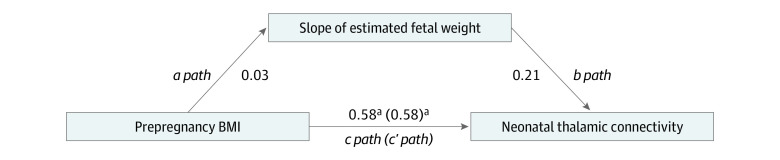
Mediation Model With Prepregnancy Body Mass Index (BMI), Estimated Fetal Weight, and Neonatal Thalamic Connectivity ^a^*P* < .001.

No significant correlation was found between EFW and the seed thalamic brain region (*r* = 0.218; 95% CI, −0.078 to 0.482; *P* = .20). Mediation analyses indicated no significant indirect association of prepregnancy BMI on neonatal thalamic connectivity through EFW (standardized coefficient = 0.005; 95% CI, −0.12 to 0.10; *P* = .93). The full mediation model is presented in Figure 4.

There was also no significant correlation of fetal head circumference with the seed thalamic brain region (*r* = −0.013; 95% CI, −0.302 to 0.284; *P* = .94). We did not conduct mediation analyses between prepregnancy BMI, fetal head circumference, and neonatal thalamic connectivity because head circumference had no significant bivariate associations with either of these variables.

## Discussion

The goal of this prospective study was to determine whether maternal prepregnancy BMI was associated with measures of fetal growth and neonatal brain functional connectivity. Prepregnancy BMI had a positive association with the slope of EFW but not fetal head circumference, an index of early brain growth. While far from definitive, maternal prepregnancy or pregnancy BMI has been associated with greater fetal physical or head size in women with obesity compared with women with reference weight,^1,35,36^ broadly consistent with our findings. In the neonatal period, prepregnancy BMI was associated with greater local thalamic connectivity and lower frontothalamic connectivity. These results suggest that maternal BMI may contribute prenatally to the regulation of offspring body weight and thalamic brain connectivity.

Our finding that higher maternal BMI was associated with altered functional connectivity in infant offspring is consistent with 2 prior studies^20,21^ of functional connectivity in women with obesity compared with women with reference weight, although significant findings were reported in different brain regions and not in the thalamus. Only recently have studies with adult humans and rodents demonstrated that thalamic connectivity is involved in reward and cognitive control processing, which are brain systems integral to regulating feeding behavior.^37,38,39^ These reports identified that neurons in the paraventricular thalamic nucleus receive input from the hypothalamus and nucleus accumbens and mediate both feeding behavior and regulation of body weight.^38^ To our knowledge, our findings are the first to demonstrate the association of maternal prepregnancy BMI with thalamic connectivity in human infants. Projections from the paraventricular thalamic nucleus to the prefrontal cortex subserve reward, motivation, and decision-making processes.^37,38^ Given the associations of lower frontothalamic connectivity with increasing maternal BMI, future studies that consider behavioral tasks related to delay of gratification and food diaries during infancy may help us better understand the functional associations of these connections in the developing brain. Indeed, our findings suggest a new brain target for animal models of maternal BMI. Previous models have shown that higher maternal BMIs lead to reduced or shortened neuron size in the hippocampus^40,41^ and deficiencies in neuropeptides in the hypothalamus^42^ in offspring. Cellular and molecular studies focused on the effects of maternal BMI on the developing offspring thalamus may elucidate the underlying cellular basis for our observed thalamic connectivity findings.

Potential maternal biological mechanisms that may influence the association of maternal prepregnancy BMI with fetal growth and brain development could be the activation of the maternal immune (eg, inflammation) or endocrine (eg, cortisol) systems.^43,44^ In addition, a 2020 study by Freeman^45^ found that these 2 systems may have sex-specific associations, as Freeman identified that in the context of prenatal depression, there were higher levels of maternal inflammation (as measured by C-reactive protein) in women pregnant with male fetuses and higher levels of cortisol in women pregnant with female fetuses.

Our study has several features that enhance understanding of the associations of maternal prepregnancy BMI with fetal growth parameters. First, our sample had no women with comorbid medical disorders. As adolescents are less likely to have chronic medical conditions that are often comorbid with higher BMI, they provide a good model for isolating the association of BMI with infant outcomes. Moreover, as most studies report findings in the context of maternal diabetes or, in the case of rodent studies, a high-fat diet,^46,47^ our study suggests that maternal prepregnancy BMI and other health-related factors can have independent associations with fetal development.^1^ Second, our study considers the full distribution of maternal BMI values. As many prior studies compare dichotomized weight categories,^20,21^ it is difficult to determine whether their findings generalize to the continuum of BMI or are specific to obesity. Third, EHR reviews provided parameters of fetal growth and the opportunity to consider multiple indices of fetal development in association with maternal BMI and infant brain connectivity measures. We calculated growth estimates across gestation, rather than creating separate curves for each trimester, as was done previously.^28^ Pooling data points across gestation provides more stable growth curves by increasing the number of observations per infant.

### Limitations

Our study has several limitations. The study sample is representative of the Washington Heights district of New York, New York, a primarily Hispanic community, but is not representative of national demographic characteristics. The findings therefore may not generalize to the national population. Our maternal sample consisted of adolescent and young adult women, and so the observed associations may not generalize to pregnant women of older ages. Our sample size likely limited our statistical power to detect mediation associations among BMI, fetal growth, and neonatal MRI measures. The presence of eating disorders (eg, bulimia) was not assessed in the study, which could relate to maternal BMI. Infant nutrition (eg, breastfeeding) was not assessed. Infant feeding practices and nutrition should be assessed in future studies to determine whether they are associated with fetal brain development. Despite the longitudinal nature of our study, we have only a single point for infant imaging. Developmental trajectories of functional connectivity measures may have provided a better assessment of the associations of maternal prepregnancy BMI with the developing brain.^48^

## Conclusions

Maternal BMI is an index associated with physical and nutritional health. Our findings suggest that BMI is an important prenatal exposure measure to consider in infant development, given that BMI was found to be associated with fetal weight gain and neonatal brain connectivity measures. Future studies that include behavioral measures (eg, observational and parent scales of frequency, rate, and amount of feeding) at the same age that neuroimaging data are collected may further specify how altered brain development is associated with behavioral disturbances in offspring. This study could serve as a beginning point for future intervention studies to determine whether improving maternal BMI may be associated with fetal brain development to optimize circuit development in areas key to regulating nutrient intake and satiety signals^49^ and subsequently support optimal long-term infant cognitive, social, and behavioral capacities.

## Notes

Supplement. **eAppendix.** Supplemental Methods **eTable.** Association of Prepregnancy Body Mass Index With Fetal Head Circumference and Estimated Fetal Weight
