# Supplementary material for: Association of Maternal Prepregnancy Body Mass Index With Fetal Growth and Neonatal Thalamic Brain Connectivity Among Adolescent and Young Women
Source: JAMA Netw Open. 2020 Nov 3;3(11):e2024661. doi: 10.1001/jamanetworkopen.2020.24661 (PMC7610195; doi:10.1001/jamanetworkopen.2020.24661)
Supplement: Supplement. — eAppendix. Supplemental Methods eTable. Association of Prepregnancy Body Mass Index With Fetal Head Circumference and Estimated Fetal Weight [file jamanetwopen-e2024661-s001.pdf]

Supplemental Online Content

Spann MN, Scheinost D, Feng T, et al. Association of maternal prepregnancy body mass index with fetal growth and neonatal thalamic brain connectivity among adolescent and young women. *JAMA Netw Open*. 2020;3(11):e2024661. doi:10.1001/jamanetworkopen.2020.24661

eAppendix. Supplemental Methods

eTable. Association of Prepregnancy Body Mass Index With Fetal Head Circumference and Estimated Fetal Weight

This supplemental material has been provided by the authors to give readers additional information about their work.

© 2020 Spann MN et al. *JAMA Network Open.*

eAppendix. Supplemental Methods

Race/Ethnicity Classification. Race and ethnicity categories are defined by the U.S. census (https://2020census.gov/en/about-questions/2020-census-questions-race.html). Race categories included White, black or African American, American Indian or Alaska Native, Asian, Native Hawaiian or Pacific Islander, and Other. Ethnicity categories included Hispanic/Latina and Non-Hispanic/Latina. Participants completed surveys during in-person sessions on an iPad using RedCap software and self-reported their race and ethnicity. Race and ethnicity was collected as part of standard demographic information obtained in the study.

Imaging Parameters High resolution anatomical T2-weighted images were acquired using a 2D, multiple-shot, fast spin echo pulse sequence that employed PROPELLER (Periodically Rotated Overlapping Parallel Lines with Enhanced Reconstruction) to reduce motion artifacts in reconstructed MR images (Pipe, 1999): repetition time (TR)=10,000 ms; echo time (TE)=130 ms; echo train length (ETL)=32; matrix size=192×192; field of view (FOV)=190×190 mm; phase FOV=100%; slice thickness=1.0 mm; number of excitations (NEX)=2. The spatial resolution of the T2-weighted images was 1mm3. Functional images were acquired using a standard echo-planar imaging sequence: TR=2,200 ms; TE=30 ms; matrix size=64×64; FOV=190×190 mm; phase FOV=100%; slice thickness=5.0 mm, contiguous; number of slices=24; bandwidth=7812.5 Hz. Although the number of runs acquired varied per participant due to compliance, a median of six runs of 102 volumes (3 minutes 44.4 sec each) were obtained for each infant.

Motion Analysis Given the technical challenges of scanning neonates, every participant had a different number of resting-state runs. For example, runs were stopped if the infant awoke, significantly moved, or cried during the run. As motion and amount of data for analysis affects functional connectivity measures (Van Dijk et al., 2012; Noble et al., 2017), we employed a strict inclusion criterion that participants had at least 2 runs of data with an average frame-to-frame motion of <0.1 mm. The median number of partial or full runs collected per participant was 6, with a range of 2-12 runs. The median number of runs removed was 2, with a range of 0-8 runs, leaving a median of 3 low motion runs per participant, with a range of 2-7 runs. Therefore, we required a minimum of 2 low motion runs to retain the maximum number of participants while still having >5 minutes of resting-state data available from each infant for analysis. For infants with more than 2 runs with acceptable movement, we selected the 2 runs with the least frame-to-frame motion. Further, as described in the main text, we employed global signal regression, regression of a 24-parameter motion model, and spatial uniform smoothing (FWHM=8 mm) to minimize motion confounds not accounted for by our inclusion criteria. Finally, given our strict motion inclusion criteria of an average frame-to-frame displacement <0.1 mm, only an average of 2.1 frames per participant had motion greater than the recommended censoring threshold of 0.2 mm. Post-hoc analysis on censored data or covarying for frame-to-frame displacement, did not change the imaging results.

© 2020 Spann MN et al. *JAMA Network Open.*

eTable. Association of Pre-Pregnancy Body Mass Index with Fetal Head Circumference and Estimated Fetal Weight

	**Unadjusted**			**Adjusted[a]**			**Adjusted[b]**			**Adjusted[c]**		
	T	*p*	CI	T	*P*	CI	T	*p*	CI	T	*p*	CI
Fetal Head Circumference	-0.39	0.70	-0.02-0.02	-0.09	0.93	-0.02-0.02	-0.34	0.73	-0.03-0.02	-0.58	0.56	-0.03-0.01
Estimated Fetal Weight	2.59	0.01**	0.16-0.18	2.07	0.04*	0.02-1.11	2.9	0.005**	0.26-1.38	2.51	0.01**	0.14-1.17

Adjusted for:
[a] Maternal age, type of delivery, and pregnancy complications
[b] Maternal pregnancy weight gain
[c] Infant sex
*p<.05; **p<.01; ***p<.001

© 2020 Spann MN et al. *JAMA Network Open.*
